# Supplementary material for: Coupling of a Novel TIMP3 Peptide to Carboxypeptidase G2 for Pro-Drug Activation at the Tumour Site
Source: Molecules. 2021 Jan 25;26(3):625. doi: 10.3390/molecules26030625 (PMC7865317; doi:10.3390/molecules26030625)
Supplement: Supplementary file 1 [file molecules-26-00625-s001.pdf]

Supplement data

Sequence: CPG2 codon optimised no periplasmic seq + His tag single AgeI Range: 1 to 1223

```
   >NdeI
     |
     | 10        20        30        40        50        60        70        80        90
CATATGGCGCTGGCCCAGAAACGTGATAACGTGCTGTTTCAGGCGGCGACCGATGAACAGCCGGCGGTGATTAAAACGCTGGAAAAACTG
GTATACCGCGACCGGGTCTTTGCACTATTGCACGACAAAGTCCGCCGCTGGCTACTTGTCGGCCGCCACTAATTTTGCGACCTTTTTGAC
   M  A  L  A  Q  K  R  D  N  V  L  F  Q  A  A  T  D  E  Q  P  A  V  I  K  T  L  E  K  L>
   ________________________________________CDS_________________________________________>

                >AgeI
                  |
        100       110 |     120       130       140       150       160       170       180
GTGAACATTGAAACCGGCACCGGTGATGCGGAAGGTATTGCGGCAGCGGGTAACTTTCTGGAAGCGGAACTGAAAAACCTGGGCTTTACC
CACTTGTAACTTTGGCCGTGGCCACTACGCCTTCCATAACGCCGTCGCCCATTGAAAGACCTTCGCCTTGACTTTTTGGACCCGAAATGG
 V  N  I  E  T  G  T  G  D  A  E  G  I  A  A  A  G  N  F  L  E  A  E  L  K  N  L  G  F  T>
___________________________________________CDS_____________________________________________>

        190       200       210       220       230       240       250       260       270
GTGACCCGTAGCAAAAGCGCGGGTCTGGTGGTGGGCGATAACATTGTGGGCAAAATTAAAGGCCGTGGCGGTAAAAACCTGCTGCTGATG
CACTGGGCATCGTTTTCGCGCCCAGACCACCACCCGCTATTGTAACACCCGTTTTAATTTCCGGCACCGCCATTTTTGGACGACGACTAC
 V  T  R  S  K  S  A  G  L  V  V  G  D  N  I  V  G  K  I  K  G  R  G  G  K  N  L  L  L  M>
___________________________________________CDS_____________________________________________>

        280       290       300       310       320       330       340       350       360
AGCCACATGGATACCGTGTATCTGAAAGGCATTCTGGCCAAAGCGCCGTTTCGTGTGGAAGGCGATAAAGCGTATGGCCCGGGTATTGCG
TCGGTGTACCTATGGCACATAGACTTTCCGTAAGACCGGTTTCGCGGCAAAGCACACCTTCCGCTATTTCGCATACCGGGCCCATAACGC
 S  H  M  D  T  V  Y  L  K  G  I  L  A  K  A  P  F  R  V  E  G  D  K  A  Y  G  P  G  I  A>
___________________________________________CDS_____________________________________________>

        370       380       390       400       410       420       430       440       450
GATGATAAAGGCGGCAACGCGGTGATTCTGCATACCCTGAAACTGCTGAAAGAATATGGCGTGCGTGATTATGGCACCATTACCGTGCTG
CTACTATTTCCGCCGTTGCGCCACTAAGACGTATGGGACTTTGACGACTTTCTTATACCGCACGCACTAATACCGTGGTAATGGCACGAC
 D  D  K  G  G  N  A  V  I  L  H  T  L  K  L  L  K  E  Y  G  V  R  D  Y  G  T  I  T  V  L>
___________________________________________CDS_____________________________________________>

        460       470       480       490       500       510       520       530       540
TTTAACACCGATGAAGAAAAAGGCAGCTTTGGCAGCCGTGATCTGATTCAGGAAGAAGCGAAACTGGCCGATTATGTTCTGAGCTTTGAA
AAATTGTGGCTACTTCTTTTTCCGTCGAAACCGTCGGCACTAGACTAAGTCCTTCTTCGCTTTGACCGGCTAATACAAGACTCGAAACTT
 F  N  T  D  E  E  K  G  S  F  G  S  R  D  L  I  Q  E  E  A  K  L  A  D  Y  V  L  S  F  E>
___________________________________________CDS_____________________________________________>
```

```
        550        560        570        580        590        600        610        620        630
CCGACCAGCGCGGGTGATGAAAAACTGAGCCTGGGCACCAGCGGCATTGCGTATGTGCAGGTGAACATTACCGGCAAAGCGAGCCATGCG
GGCTGGTCGCGCCCACTACTTTTTGACTCGGACCCGTGGTCGCCGTAACGCATACACGTCCACTTGTAATGGCCGTTTCGCTCGGTACGC
 P  T  S  A  G  D  E  K  L  S  L  G  T  S  G  I  A  Y  V  Q  V  N  I  T  G  K  A  S  H  A>
___________________________________________CDS___________________________________________>

        640        650        660        670        680        690        700        710        720
GGTGCGGCACCGGAACTGGGTGTGAACGCGCTGGTTGAAGCGAGCGATCTGGTGCTGCGTACCATGAACATTGATGATAAAGCGAAAAAC
CCACGCCGTGGCCTTGACCCACACTTGCGCGACCAACTTCGCTCGCTAGACCACGACGCATGGTACTTGTAACTACTATTTCGCTTTTTG
 G  A  A  P  E  L  G  V  N  A  L  V  E  A  S  D  L  V  L  R  T  M  N  I  D  D  K  A  K  N>
___________________________________________CDS___________________________________________>

        730        740        750        760        770        780        790        800        810
CTGCGTTTTAACTGGACCATTGCGAAAGCGGGCAACGTGAGCAACATTATTCCGGCGAGCGCGACCCTGAATGCGGATGTGCGTTATGCG
GACGCAAAATTGACCTGGTAACGCTTTCGCCCGTTGCACTCGTTGTAATAAGGCCGCTCGCGCTGGGACTTACGCCTACACGCAATACGC
 L  R  F  N  W  T  I  A  K  A  G  N  V  S  N  I  I  P  A  S  A  T  L  N  A  D  V  R  Y  A>
___________________________________________CDS___________________________________________>

        820        830        840        850        860        870        880        890        900
CGTAACGAAGATTTTGATGCGGCCATGAAAACGCTGGAAGAACGTGCGCAGCAGAAAAAACTGCCGGAAGCGGATGTGAAAGTGATTGTG
GCATTGCTTCTAAAACTACGCCGGTACTTTTGCGACCTTCTTGCACGCGTCGTCTTTTTTGACGGCCTTCGCCTACACTTTCACTAACAC
 R  N  E  D  F  D  A  A  M  K  T  L  E  E  R  A  Q  Q  K  K  L  P  E  A  D  V  K  V  I  V>
___________________________________________CDS___________________________________________>

        910        920        930        940        950        960        970        980        990
ACCCGTGGCCGTCCGGCGTTTAACGCGGGCGAAGGCGGCAAAAAACTGGTGGATAAAGCGGTGGCGTATTATAAAGAAGCAGGCGGCACC
TGGGCACCGGCAGGCCGCAAATTGCGCCCGCTTCCGCCGTTTTTTGACCACCTATTTCGCCACCGCATAATATTTCTTCGTCCGCCGTGG
 T  R  G  R  P  A  F  N  A  G  E  G  G  K  K  L  V  D  K  A  V  A  Y  Y  K  E  A  G  G  T>
___________________________________________CDS___________________________________________>

       1000       1010       1020       1030       1040       1050       1060       1070       1080
CTGGGTGTGGAAGAACGTACCGGCGGTGGCACCGATGCGGCGTATGCGGCCCTGAGCGGCAAGCCGGTGATTGAAAGCCTGGGCCTGCCG
GACCCACACCTTCTTGCATGGCCGCCACCGTGGCTACGCCGCATACGCCGGGACTCGCCGTTCGGCCACTAACTTTCGGACCCGGACGGC
 L  G  V  E  E  R  T  G  G  G  T  D  A  A  Y  A  A  L  S  G  K  P  V  I  E  S  L  G  L  P>
___________________________________________CDS___________________________________________>

       1090       1100       1110       1120       1130       1140       1150       1160       1170
GGCTTTGGCTATCATAGCGATAAAGCGGAATATGTGGATATTAGCGCGATTCCGCGTCGTCTGTATATGGCGCGTCGTCTGATTATGGAT
CCGAAACCGATAGTATCGCTATTTCGCCTTATACACCTATAATCGCGCTAAGGCGCAGCAGACATATACCGCGCAGCAGACTAATACCTA
 G  F  G  Y  H  S  D  K  A  E  Y  V  D  I  S  A  I  P  R  R  L  Y  M  A  R  R  L  I  M  D>
___________________________________________CDS___________________________________________>

                                             >HindIII
```

```
                                            |
      1180      1190      1200      1210    | 1220
CTGGGTGCGGGCAAACATCATCACCATCATCATTAATAAAAGCTTGCGGCCGC
GACCCACGCCCGTTTGTAGTAGTGGTAGTAGTAATTATTTTCGAACGCCGGCG
 L  G  A  G  K  H  H  H  H  H  H  *>
________________CDS_________________>
```

Sequence: p700 for CPG2 modified for synthesis Range: 1 to 222

```
   >NdeI
      |
      | 10        20        30        40        50        60        70        80        90
CATATGAAAATCAAATCCTGCTACTATCTGCCGTGTTTCGTAACCAGCAAGAATGGCGGTGGTGGCTCAGGTGGAGGCGGCAGTGGCGGC
GTATACTTTTAGTTTAGGACGATGATAGACGGCACAAAGCATTGGTCGTTCTTACCGCCACCACCGAGTCCACCTCCGCCGTCACCGCCG
       K  I  K  S  C  Y  Y  L  P  C  F  V  T  S  K  N>
      _____________________P700______________________>
                                                       G  G  G  G  S  G  G  G  G  S  G  G>
                                                      _________[GLY4SER]4 LINKER__________>

       100       110       120       130       140       150       160       170       180
GGTGGTTCTGGCGGAGGTGGGTCGGCATTAGCGCAGAAACGCGATAACGTTCTGTTTCAGGCTGCGACTGACGAACAACCAGCCGTCATT
CCACCAAGACCGCCTCCACCCAGCCGTAATCGCGTCTTTGCGCTATTGCAAGACAAAGTCCGACGCTGACTGCTTGTTGGTCGGCAGTAA
 G  G  S  G  G  G  G  S>
___[GLY4SER]4 LINKER____>
                         A  L  A  Q  K  R  D  N  V  L  F  Q  A  A  T  D  E  Q  P  A  V  I>
                        ______________________________CPG2________________________________>

                                      >AgeI
                                         |
       190       200       210       220 |
AAAACGTTGGAGAAACTGGTGAACATTGAAACCGGGACCGGT
TTTTGCAACCTCTTTGACCACTTGTAACTTTGGCCCTGGCCA
 K  T  L  E  K  L  V  N  I  E  T  G  T  G>
__________________CPG2___________________>
```

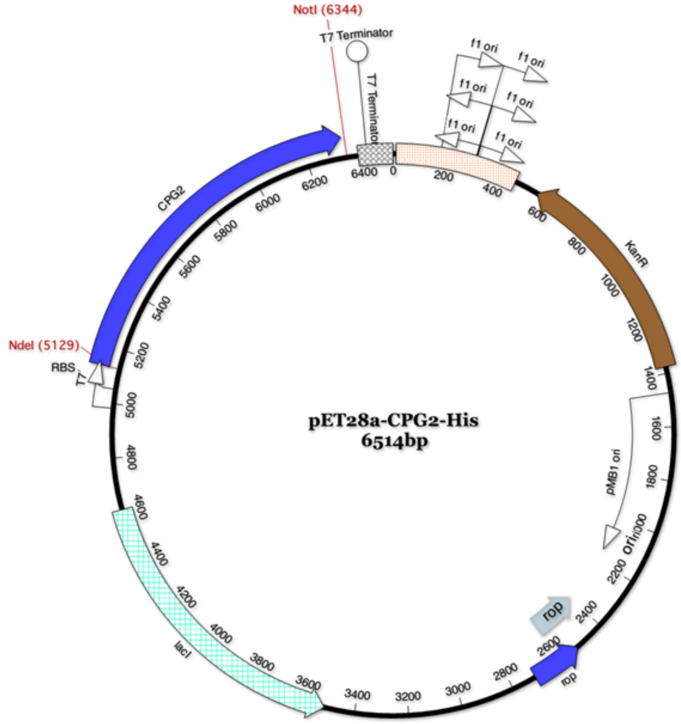

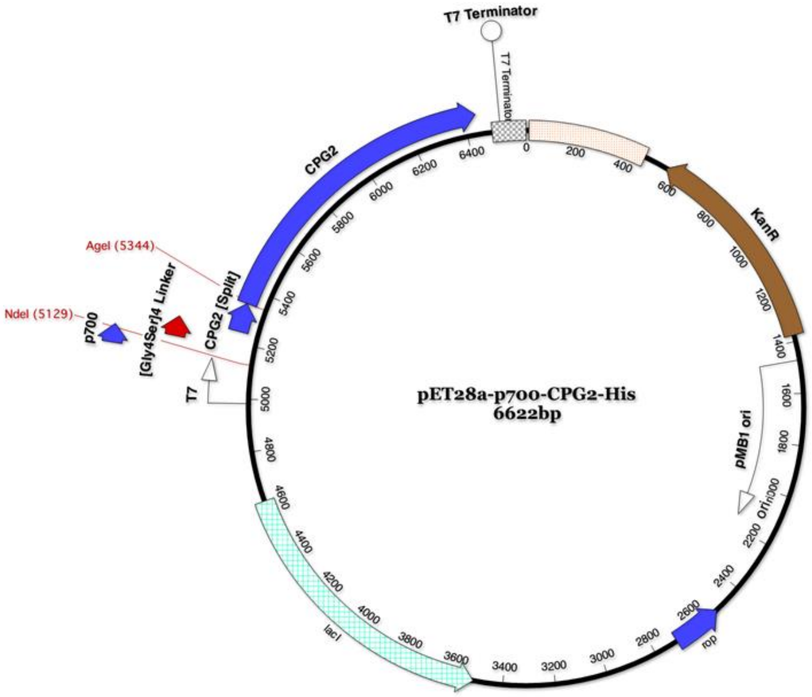

Figure A1: Insertion of codon optimised CPG2 and CPG2-p700 into pET28a plasmid vector

CPG2 (long blue) inserted between NdeI and NotI sites while p700 plus linker (short blue and short red) are inserted at AgeI and NdeI sites to align the sequences in a correct open reading frame for a codon optimised vector.
